# Rates of immune cell infiltration in patients with triple-negative breast cancer by molecular subtype

**DOI:** 10.1371/journal.pone.0204513

**Published:** 2018-10-12

**Authors:** Kenichi Harano, Ying Wang, Bora Lim, Robert S. Seitz, Stephan W. Morris, Daniel B. Bailey, David R. Hout, Rachel L. Skelton, Brian Z. Ring, Hiroko Masuda, Arvind U. K. Rao, Steven Van Laere, Francois Bertucci, Wendy A. Woodward, James M. Reuben, Savitri Krishnamurthy, Naoto T. Ueno

**Affiliations:** 1 Morgan Welch Inflammatory Breast Cancer Research Program and Clinic, The University of Texas MD Anderson Cancer Center, Houston, Texas, United States of America; 2 Department of Breast Medical Oncology, The University of Texas MD Anderson Cancer Center, Houston, Texas, United States of America; 3 Department of Pulmonology Medicine and Oncology, Graduate School of Medicine, Nippon Medical School, Bunkyo-ku, Tokyo, Japan; 4 Department of Bioinformatics and Computational Biology, The University of Texas MD Anderson Cancer Center, Houston, Texas, United States of America; 5 Insight Genetics, Inc., Nashville, Tennessee, United States of America; 6 College of Life Science, Huazhong University of Science and Technology, Wuhan, China; 7 Department of Breast Surgical Oncology, Showa University, Shinagawa-ku, Tokyo, Japan; 8 Department of Radiation Oncology, The University of Texas MD Anderson Cancer Center, Houston, Texas, United States of America; 9 Center for Oncological Research, Faculty of Medicine and Health Sciences, University of Antwerp, Antwerp, Belgium; 10 Predictive Oncology team, CRCM, Institut Paoli-Calmettes, Marseille, France; 11 Department of Hematopathology, The University of Texas MD Anderson Cancer Center, Houston, Texas, United States of America; 12 Department of Pathology, The University of Texas MD Anderson Cancer Center, Houston, Texas, United States of America; University of South Alabama Mitchell Cancer Institute, UNITED STATES

## Abstract

In patients with triple-negative breast cancer (TNBC), tumor-infiltrating lymphocytes (TILs) are associated with improved survival. Lehmann et al. identified 4 molecular subtypes of TNBC [basal-like (BL) 1, BL2, mesenchymal (M), and luminal androgen receptor (LAR)], and an immunomodulatory (IM) gene expression signature indicates the presence of TILs and modifies these subtypes. The association between TNBC subtype and TILs is not known. Also, the association between inflammatory breast cancer (IBC) and the presence of TILs is not known. Therefore, we studied the IM subtype distribution among different TNBC subtypes. We retrospectively analyzed patients with TNBC from the World IBC Consortium dataset. The molecular subtype and the IM signature [positive (IM+) or negative (IM-)] were analyzed. Fisher’s exact test was used to analyze the distribution of positivity for the IM signature according to the TNBC molecular subtype and IBC status. There were 88 patients with TNBC in the dataset, and among them 39 patients (44%) had IBC and 49 (56%) had non-IBC. The frequency of IM+ cases differed by TNBC subtype (p = 0.001). The frequency of IM+ cases by subtype was as follows: BL1, 48% (14/29); BL2, 30% (3/10); LAR, 18% (3/17); and M, 0% (0/21) (in 11 patients, the subtype could not be determined). The frequency of IM+ cases did not differ between patients with IBC and non-IBC (23% and 33%, respectively; p = 0.35). In conclusion, the IM signature representing the underlying molecular correlate of TILs in the tumor may differ by TNBC subtype but not by IBC status.

## Introduction

Triple-negative breast cancer (TNBC) accounts for 10% to 20% of breast cancers. TNBC is an aggressive tumor, and patients with TNBC have a higher risk of both local and distant recurrence compared to patients with other types of breast cancer [1]. Patients with TNBC have higher rates of pathological complete response (pCR) following neoadjuvant chemotherapy than patients with other types of breast cancer, but TNBC patients without a pCR have a markedly worse prognosis than TNBC patients with a pCR [2].

Several studies have shown that in patients with TNBC, there is a linear relationship between the number of tumor-infiltrating lymphocytes (TILs), mononuclear immune cells that infiltrate tumor, and recurrence-free survival [3–5]. It has also been reported that in patients with breast cancer, the presence of TILs is associated with increased rates of pCR following neoadjuvant chemotherapy [6, 7]. Thus, TILs are a prognostic factor and predictor of response to cytotoxic chemotherapy in patients with TNBC.

TNBCs are heterogeneous. Lehmann et al. reported in 2011 that TNBCs could be grouped into 6 molecular subtypes: basal-like (BL) 1, BL2, mesenchymal (M), mesenchymal stem-like (MSL), immunomodulatory (IM), and luminal androgen receptor (LAR) [8]. They suggested that the subtypes exhibit the following characteristics: BL1, increased expression of genes associated with the cell cycle and DNA damage response; BL2, growth factor signaling; M, increased expression of epithelial-mesenchymal transition (EMT) and growth factor pathways; MSL, increased expression of EMT and growth factor pathways and decreased expression of genes involved in proliferation; IM, expression of genes encoding immune antigens and cytokines; and LAR, androgen receptor signaling. We previously reported that the TNBC subtype is a predictor of pCR after neoadjuvant chemotherapy [9]: the BL1 subtype was associated with the highest pCR rate (52%), and the BL2 and LAR subtypes had the lowest pCR rates (0% and 10%, respectively).

Recently, using laser capture microdissection and histopathological quantification, Lehmann et al. found that transcripts in the previously defined IM and MSL subtypes came from TILs and tumor-associated stromal cells, respectively, and they reduced the number of TNBC molecular subtypes to 4: BL1, BL2, M, and LAR [10]. Further, they showed that the IM gene expression signature is an indicator of the presence of TILs and incorporated the IM signature into TNBC subtyping as a modifier of the other subtypes rather than a separate subtype.

The association between TNBC subtype and the presence of TILs is not known. On the basis of the above-noted clinical and molecular data, we hypothesized that the BL1 subtype has a high rate of IM signature and that the BL2 and LAR subtypes have low rates of IM signature, which reflects immune infiltration.

In addition to being characterized by TNBC subtype, TNBC can be classified according to whether it represents inflammatory breast cancer (IBC) or non-inflammatory breast cancer (non-IBC). IBC is a relatively rare and aggressive cancer that presents with rapid onset of redness and swelling of the breast [11]. Several inflammatory signaling pathways, including NF-κB, COX-2, JAK/STAT, IL-6, tumor necrosis factor alpha, and interferon gamma, have been suggested to contribute to the tumorigenesis of IBC [12]. We previously reported that the TNBC molecular subtypes are expressed in both inflammatory and non-inflammatory TNBC and that we found no unique IBC-specific TNBC subtypes by mRNA gene expression profiling [13]. However, the association between IBC and the presence of TILs is not known. We hypothesized that IM signature is more frequent in patients with IBC than in those with non-IBC.

To test our hypotheses, we studied gene expression in patients with TNBC with IBC and non-IBC and evaluated the relationship between TNBC molecular subtype and IM signature and also the relationship between IBC status and IM signature.

## Methods

### Patients

We retrospectively analyzed gene expression profiles and clinical data of patients with TNBC with known IBC status from the World IBC Consortium dataset [14]. Three institutions contributed to this dataset: The University of Texas MD Anderson Cancer Center, Houston, TX; General Hospital Sint-Augustinus, Antwerp, Wilrijk, Belgium; and Institut Paoli-Calmettes, Marseille, France. We obtained clinical data (patient age, stage, histologic subtype, grade, and treatment) and gene expression profiles from the 3 institutes. For the World IBC Consortium database study, patients at each site gave informed consent for voluntary participation, and the study was approved by the institutional review boards of the 3 participating centers. IBC was identified according to the consensus diagnostic criteria [11, 14, 15]. At each of the 3 centers, the diagnostic criteria for the diagnosis of IBC were as follows: 1) rapid onset of breast erythema and/or peau d’orange and/or warm breast with or without an underlying palpable mass, 2) duration of clinical symptoms/signs no more than 6 months, 3) erythema occupying at least one-third of the breast, and 4) pathological confirmation of invasive carcinoma. TNBC was diagnosed according to gene expression profiling. The original gene expression profiling and identification of TNBC cases has already been reported in our previous paper [13]. For gene expression profiling, all samples were run on the Affymetrix HGU133 platform.

TNBCtype (Insight Genetics, Inc., Nashville, TN, USA) was used to assign TNBC subtypes. TNBCtype is a new algorithm for TNBC subtyping that reduces gene signatures from the original 2188 genes described by Lehmann et al. [8] to 101 genes including control housekeeping genes in order to optimize gene expression profiles [16, 17]. The optimized TNBCtype algorithm is designed to classify TNBC according to 1 of the 4 intrinsic subtypes and then define TNBC tumors as positive (IM+) or negative (IM-) for the IM gene expression signature. It has been shown that IM gene expression signature is correlated with the level of TILs in the tumor specimen [10]. TNBC tumors classified as IM+ are highly enriched for genes involved in immune cell processes, including immune cell signaling, cytokine signaling, antigen processing and presentation, and signaling through core immune signal transduction pathways. Using TNBCtype, we grouped patients by the 4 subtypes (BL1, BL2, M, and LAR), then identified whether each case of TNBC was positive or negative for the IM signature.

The study reported herein was approved by the Institutional Review Board of MD Anderson Cancer Center (protocol number PA15-0954). The Institutional Review Board waived the requirement for informed consent because this study was a retrospective data review that involved no diagnostic or therapeutic intervention and no direct patient contact.

### Statistical analysis

We constructed 4×2 (molecular subtype *versus* positive or negative for IM signature) or 2×2 (IBC status *versus* positive or negative for IM signature) contingency tables and compared the distributions using Fisher’s exact test. A p value of <0.05 was considered to indicate a significant association.

## Results

In total, 88 TNBC patients (39 with IBC and 49 with non-IBC) were included in this study. The patient characteristics are summarized in Table 1. Of the 88 TNBC patients in this study, 55 (63%) had stage III or IV disease, and 39 (44%) had IBC. All patients received multidisciplinary treatment according to treatment guidelines of each hospital. Among the 71 patients with stage I-III disease, 21 (78%) of the 27 IBC patients and 29 (66%) of the 44 non-IBC patients received neoadjuvant chemotherapy. The neoadjuvant chemotherapy regimen consisted of an anthracycline-based plus taxane-based chemotherapy.

**Table 1:** Patient characteristics.

Variable	N	%
Total number of patients	88	
Age, median (range), years	52 (26–78)	
Stage		
I	9	10
II	17	19
III	45	51
IV	10	12
Unknown	7	8
IBC status		
Non-IBC	49	56
IBC	39	44
Histology		
Invasive ductal carcinoma	80	91
Invasive lobular carcinoma	4	5
Other	4	5
Nuclear grade		
I	3	3
II	14	16
III	68	77
Unknown	3	3
Neoadjuvant chemotherapy (for stage I-III disease, 71 patients)		
Received	50	70
Not received	20	28
Unknown	1	2

IBC, inflammatory breast cancer.

The distribution of IM signature by intrinsic subtype is shown in Table 2. Among the 88 patients with TNBC, 29 had BL1, 10 had BL2, 21 had M, and 17 had LAR subtype, while 11 did not have a clear subtype. In total, 25 patients (28%) had IM+ and 63 (72%) had IM- TNBC. The frequency of IM+ cases differed significantly by molecular subtype (p = 0.00036). The frequency of IM+ cases was higher in the BL1 subtype (14 of 29, 48%) than in other subtypes. No IM+ cases were observed in the M subtype, and only 3 of 17 (18%) cases of LAR subtype were IM+.

**Table 2:** Distribution of IM signature in patients with TNBC by molecular subtype and IBC status.

Subgroup	Total No. of patients	No. (%) IM+	No. (%) IM-	*p* value
	(n = 88)	(n = 25)	(n = 63)	
Subtype				
BL1	29	14 (48)	15 (52)	0.00036
BL2	10	3 (30)	7 (70)	
M	21	0 (0)	21 (100)	
LAR	17	3 (18)	14 (82)	
ND	11	5 (45)	6 (55)	
IBC status				
IBC	39	9 (23)	30 (77)	0.35
Non-IBC	49	16 (33)	33 (67)	

IM, immunomodulatory; TNBC, triple-negative breast cancer; IBC, inflammatory breast cancer; BL, basal-like; M, mesenchymal; LAR, luminal androgen receptor; ND, not determined.

The distribution of IM signature in patients with IBC and non-IBC is also shown in Table 2. IM+ cases occurred at roughly the same frequency in patients with IBC (23%) and non-IBC (33%) (p = 0.35).

## Discussion

We found that TNBC molecular subtypes differed with respect to the proportion of cases with the IM signature. Forty-eight percent of the BL1-subtype TNBCs were positive for the IM signature, whereas the signature was not observed in TNBCs of M subtype and was found in only 18% of TNBCS of LAR subtype. The distribution of the IM signature did not differ between patients with IBC and non-IBC.

Recent studies have shown significant correlation between TILs and clinical outcomes in TNBC. Several studies showed that a higher number of TILs was associated with improved recurrence-free survival [3–5]. Further, the presence of TILs has been associated with greater benefit from neoadjuvant chemotherapy in patients with breast cancer. For example, in the GeparDuo and GeparTrio clinical trials of neoadjuvant chemotherapy with an anthracycline and a taxane, the pCR rate was significantly higher in patients with tumors in which TILs accounted for at least 60% of the tumor stromal area than in patients with tumors in which TILs accounted for less than 60% [6].

To our knowledge, the association between TNBC subtype and the presence of TILs has previously been examined only in a single study performed by Lehmann et al. [10]. We previously reported that TNBC molecular subtype predicts pCR status. The BL1 subtype had the highest pCR rate (52%), while the BL2 and LAR subtypes had the lowest pCR rates (0% and 10%, respectively) [9]. However, it has not been known why the pCR rate differs by TNBC subtype. In the current study, the BL1 subtype had a high rate of the IM+ signature (48%), while the BL2 (30%) and LAR (18%) subtypes had lower rates of the IM+ signature. These results, which are consistent with those reported by Lehmann et al. [10], suggest that the degree of immune cell infiltration and the IM signature status of tumors, which reflects this infiltration, are influenced by subtype and that immune infiltration affects the response to chemotherapy, which may partially explain our previous finding that pCR rates differ by TNBC subtypes.

In this study, we found that no patient with TNBC M subtype had IM+ signature. The M subtype is associated with increased expression of EMT and growth factor pathways. The expression of EMT genes in the tumor microenvironment has been associated with immune suppression [18]. Dongre et al. recently reported that breast tumors that have mesenchymal features express low levels of MHC-I and high levels of PD-L1 and contain immunosuppressive cells such as regulatory T-cells, M2 macrophages, and exhausted CD8+ T-cells within their stroma [19]. Our study is consistent with this observation and further suggests that TNBC of M subtype that expresses an activation of EMT is associated with immunosuppression. One potential discrepancy concerns the relatively high chemosensitivity our previous study showed for TNBC M-subtype tumors, which had a pCR rate of 31% following neoadjuvant chemotherapy [9]. Tumors that have an immunosuppressive environment and contain EMT features are generally considered to be refractory to cytotoxic chemotherapy [20, 21]; this discordance remains unsolved.

In our analysis, the frequency of IM+ tumors did not differ significantly between TNBC patients with IBC and those with non-IBC. We initially hypothesized that the proportion of IM+ tumors would be higher in IBC than in non-IBC because several inflammatory signaling pathways have been shown to be active in IBC. The reason why our results did not support this hypothesis is unclear; however, our results are consistent with our previous report that no unique IBC-specific signature was identified by mRNA gene expression analysis [13]. There may be other, non-inflammatory molecular mechanisms that lead to the tumorigenesis of IBC. For example, it has been reported that the aggressive phenotype of IBC is associated with an enrichment of cancer stem cells [22]. The function of cancer stem cells is modulated by many signaling pathways, including IL-6/STAT3, hedgehog, WNT, and Notch. Syndecan-1 (CD138), a cell-surface heparan sulfate proteoglycan, modulates cell proliferation and growth, and it has been reported that syndecan-1 may regulate expression of the IL-6/STAT3, Notch, and EGFR signaling pathways in inflammatory TNBC [23]. IBC is characterized by the clinical appearance of inflammation; however, IBC may be characterized molecularly not by inflammatory immune cells but rather by cancer stem cells.

This study has several limitations. First, the number of patients was limited, and we could not analyze the survival outcome according to intrinsic subtypes and IM signature status. Only 10 patients (11%) in this study had BL2-subtype disease. We found a similar result in our previous study in which the same dataset was used but TNBC subtyping was done by a different algorithm; in that study, only 5 patients (5.6%) had BL2-subtype disease [13]. Second, we used the World IBC Consortium dataset, which includes many patients with advanced breast tumors. This dataset may not be representative of the general population of patients with TNBC. Third, 11 patients (12.5%) had an unclassifiable molecular subtype. This may reflect the existence of hybrid TNBCs comprising more than one subtype or be due to other unknown factors. Fourth, we only analyzed gene expression profile data and did not perform histopathological confirmation of TILs. On the basis of previously reported findings, we considered the IM gene expression signature to be an indicator of the presence of TILs [10]; however, this molecular definition is not in widespread use.

In conclusion, our study demonstrated that the rate of IM+ subtype differs according to TNBC subtype, with the highest percentage of IM+ cases seen among BL1-subtype tumors and no IM+ cases seen among M-subtype tumors. This leads us to speculate that the rate of immune infiltration differs by TNBC molecular subtype. The findings also suggest that the TNBC subtype, because of its association with IM subtype, may influence the response to chemotherapy.

## Supporting information

S1 Data TNBC IM study data. (XLSX)
